# Genetic Architecture of Body Weight Variation and GWAS-Informed Genomic Selection in Formulated Diet-Adapted Mandarin Fish (*Siniperca chuatsi*)

**DOI:** 10.3390/ijms27219433

**Published:** 2026-10-22

**Authors:** Chenfan Geng, Hailiang Song, Yinglan Xia, Xin Zhang, Shuisheng Li, Shuang Liu, Jinmeng Teng, Yong Zhang, Hongxia Hu

**Affiliations:** 1 Fisheries Science Institute, Beijing Academy of Agriculture and Forestry Sciences & Beijing Key Laboratory of Fisheries Biotechnology, Beijing 100068, China; gengchenfan@baafs.net.cn (C.G.);; 2 State Key Laboratory of Biocontrol and School of Life Sciences, Southern Marine Science and Engineering Guangdong Laboratory (Zhuhai), Guangdong Provincial Key Laboratory of Aquatic Economic Animals and Guangdong Provincial Engineering Technology Research Center for Healthy Breeding of Important Economic Fish, Sun Yat-Sen University, Guangzhou 510275, China

**Keywords:** *Siniperca chuatsi*, genomic prediction efficiency, genomic selection, SNP density, genetic improvement, formulated diet adaptation, aquaculture industry, China

## Abstract

Large variation in body weight poses a major challenge to the development and industrialization of formulated-feed aquaculture in *Siniperca chuatsi*. Breeding varieties that combine formulated-diet adaptation, rapid growth, and uniform body weight is therefore important for improving production efficiency. However, the genetic architecture of body weight and optimal genomic selection (GS) strategies remain poorly understood. Here, whole-genome resequencing was performed on 522 formulated-diet-adapted individuals. Genome-wide association study (GWAS) identified 21 significant loci, with candidate genes associated with lipid metabolism, endocrine regulation, and neuroendocrine signaling. Genomic prediction was evaluated using genome-wide single-nucleotide polymorphisms (SNPs) and GWAS-prioritized SNP panels with Bayesian and machine-learning models. Prediction accuracy increased with marker number and generally plateaued at approximately 30,000 SNPs. Random forest using GWAS-prioritized markers achieved the highest accuracy, improving prediction accuracy by 4.8% over genome-wide genomic best linear unbiased prediction (GBLUP) while using fewer markers. These findings demonstrate the potential of GWAS-informed marker prioritization to reduce marker requirements and improve GS efficiency for body weight in formulated-diet-adapted *S. chuatsi*.

## 1. Introduction

Mandarin fish (*Siniperca chuatsi*) is an economically important freshwater aquaculture species in China, valued for its rapid growth, favorable flesh quality, and high consumer acceptance; reported per-unit economic returns are more than four times those of major freshwater fish species [1,2,3]. Production has continued to rise in response to market demand, reaching approximately 603,436 t in 2025, an 11.79% increase over 2024 [4]. However, conventional production still relies heavily on live forage fish, a practice that requires substantial land and labor inputs and increases the risks of pathogen transmission and disease outbreaks, while also entailing considerable management costs [1,5,6]. Feed training offers a feasible route to formulated-diet acceptance in mandarin fish [1,7]. Nevertheless, after successful adaptation, growth performance remains highly variable among individuals, and body weight differs considerably among formulated-diet-adapted fish. The genetic basis of this variation is not well understood, constraining genetic improvement of growth under formulated-diet feeding. Clarifying the genetic basis of growth variation in formulated-diet-adapted mandarin fish and identifying individuals with high genetic potential for growth are therefore important for improving this trait through genetic selection.

Research on formulated-diet adaptation in mandarin fish has to date centered on comparing individuals that readily accept formulated diets with those that remain resistant to dietary transition, in order to elucidate the molecular basis of individual variation in feed-adaptation capacity [8,9,10]. Genomic studies have addressed the genetic basis of feeding-habit domestication. Early candidate-gene work identified polymorphisms in lipid metabolism and digestion genes, including *LPL* and *PEP*, as potential correlates of artificial-diet acceptance. Subsequent whole-genome resequencing identified 57 selective sweep regions and 149 candidate genes associated with formulated-diet domestication [8]. Epigenetic and epigenomic analyses have further implicated regulatory mechanisms. Increased DNA methylation of *TFIIF* and reduced H3K27me3 levels, accompanied by altered *EZH1* expression, were associated with formulated-diet acceptance [10]. Moreover, whole-genome bisulfite sequencing identified 5976 differentially methylated regions involving 3522 genes—including *PRKACBA*, *CREB5B*, *BDNF*, and *LPL*—with enrichment in cAMP signaling, lipid metabolism, and neural plasticity [8]. Transcriptomic and metabolomic profiling has revealed coordinated changes in retinol metabolism, glycerolipid metabolism, and unsaturated fatty acid biosynthesis; for example, expression of triacylglycerol lipase and stearoyl-CoA-related genes, together with corresponding metabolic changes, differed between formulated-diet-adapted and non-adapted individuals [10,11]. 16S rRNA profiling of the gut microbiome has shown marked differences in microbial diversity and community composition between fish fed formulated diets and those fed live prey, with *Lactococcus*, *Klebsiella*, and *Woeseia* enriched in larger formulated-diet-fed individuals and greater representation of microbial pathways related to glycan and amino acid metabolism [12]. More recently, integrated analyses of serum biochemical traits, gene expression, and gut microbiota in domestication-susceptible and domestication-resistant individuals have further characterized the physiological and molecular correlates of feed-adaptation capacity [13]. Together, these findings indicate that adaptation to formulated diets is a complex process involving coordinated changes across genomic, transcriptional, epigenetic, metabolic, and gut microbial processes. Nevertheless, the emphasis has remained largely on explaining why individuals differ in their capacity to adapt to formulated diets, rather than on the genetic basis of growth variation among individuals that have already adapted successfully.

Body weight is a complex quantitative trait governed by multiple genetic variants and environmental factors, and its genetic architecture is difficult to resolve through conventional approaches [14,15]. Chromosome-scale reference genomes and high-throughput genotyping technologies have enabled genome-wide analyses of economically important traits in mandarin fish [2,16,17]. Several GWAS have investigated growth-related traits in this species. An initial GWAS based on 5205 SLAF-seq SNPs detected 11 genome-wide significant and 37 suggestive loci across six growth traits and annotated 65 candidate genes in the surrounding regions [18]. A subsequent GWAS using 49,940 ddRAD-seq SNPs in the all-female “Dinggui I” strain identified six significant and 25 suggestive loci for five growth traits, with several candidate genes implicated in growth regulation [19]. These studies support a genetic basis for growth variation in mandarin fish and provide valuable genomic resources for genetic improvement. Existing growth-related GWAS, however, have focused mainly on conventional breeding populations and have not specifically examined body weight variation in mandarin fish that have successfully adapted to formulated diets. The genetic determinants of growth performance under formulated-diet feeding thus remain inadequately characterized, and whether genomic information can effectively predict genetic merit for body weight in formulated-diet-adapted populations is largely unresolved.

Previous studies have mainly focused on the molecular mechanisms underlying formulated-diet adaptation, whereas the genetic basis of body weight variation among fish that have already adapted successfully remains poorly understood. This study therefore aimed to elucidate the genetic architecture of body weight variation in formulated-diet-adapted mandarin fish and to develop an efficient genomic prediction strategy for selective breeding. We first performed whole-genome sequencing-based GWAS to identify body weight-associated loci and candidate genes, and then incorporated GWAS-informed SNPs into genomic prediction. SNP panels with different marker densities and multiple prediction models were evaluated by cross-validation to identify a strategy with high predictive accuracy and reduced genotyping and computational requirements. By integrating GWAS with genomic prediction, this study provides insights into the genetic basis of growth variation in formulated-diet-adapted mandarin fish and establishes a practical genomic framework for improving body weight through selective breeding.

## 2. Results

### 2.1. Substantial Individual Variation in Body Weight Under Formulated Diet

A total of 522 individuals were measured for body weight, with a mean of 487.75 g (SD = 277.59 g) and a coefficient of variation of 56.91%. Individual weights ranged from 55 to 1461.2 g, a 26.6-fold difference between the lightest and heaviest individuals, indicating substantial phenotypic variation in this formulated-diet-adapted population. The narrow-sense genomic heritability (*h*^2^) of body weight was estimated as 0.684 ± 0.081 using a GBLUP model based on the genomic relationship matrix. Table 1 summarizes these descriptive statistics and genetic parameters.

### 2.2. Whole Genome Resequencing and Population Structure Analysis

**Figure 1:**
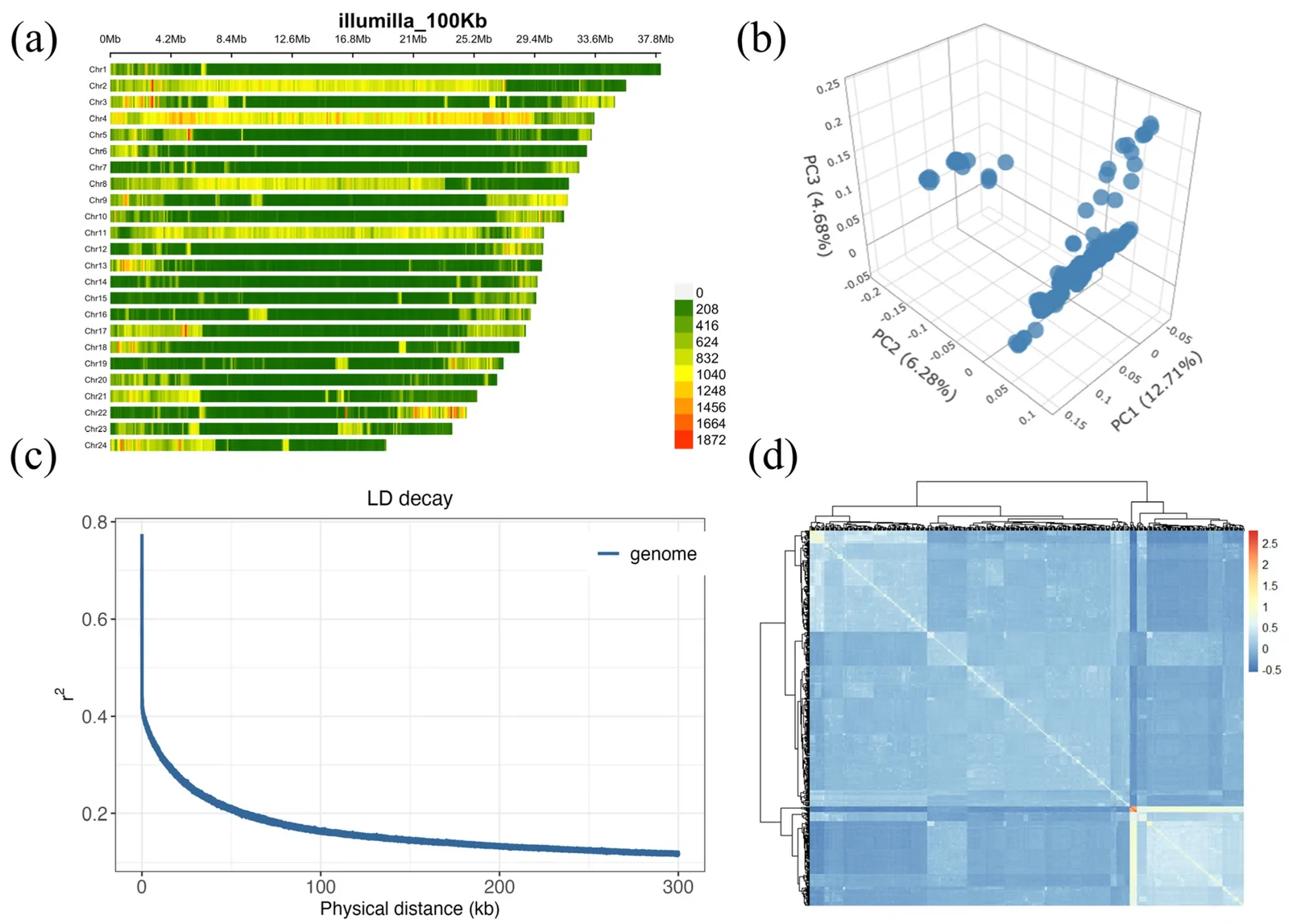
SNP distribution and population structure of mandarin fish. (**a**) Distribution of SNPs in 100 Kb windows across the genome; (**b**) principal component analyses for the first to the third dimensions of principal component (PC); (**c**) genome-wide LD decay; (**d**) heatmap of genomic relationship matrix.

Whole-genome sequencing was performed on 522 individuals. A total of 24.66 billion paired-end reads were generated, corresponding to an average of approximately 47.24 million paired-end reads per individual. The average mapping rate to the reference genome was 99.63%. Mean sequencing depth was 19.71× across all individuals, ranging from 13.65× to 29.17×. Initial variant calling identified 4,308,126 raw variants, including 2,926,398 SNPs. After stringent filtering, 2,299,387 high-quality SNPs were retained for downstream analyses. The genomic density distribution of these SNPs is shown in Figure 1a.

Principal component analysis (PCA) revealed genetic variation among the formulated-diet-accepted mandarin fish individuals, although no clear clustering pattern was observed (Figure 1b). To control for population stratification, GWAS models including the first 5 to 10 principal components (PCs) as covariates were evaluated. Among the evaluated models, inclusion of the first seven PCs resulted in the least deviation of the genomic inflation factor from 1 (***λ*** = 0.95; Supplementary Table S1), and these PCs were therefore retained in the final GWAS model. Genome-wide linkage disequilibrium (LD) decay was estimated using pairs of adjacent markers. As shown in Figure 1c, the genome-wide average squared correlation coefficient (*r*^2^) was 0.167, and the corresponding physical distance was approximately 87.91 kb. Based on this decay pattern, a 90-kb window upstream and downstream of significantly associated SNPs was used to screen for candidate genes. The heatmap of the genomic relationship matrix (G matrix) for the 522 individuals (Figure 1d) showed prominent block-like aggregation consistent with the PCA results, reflecting the population structure and providing a suitable framework for genomic prediction.

### 2.3. Genome-Wide Association Analysis of Body Weight

**Figure 2:**
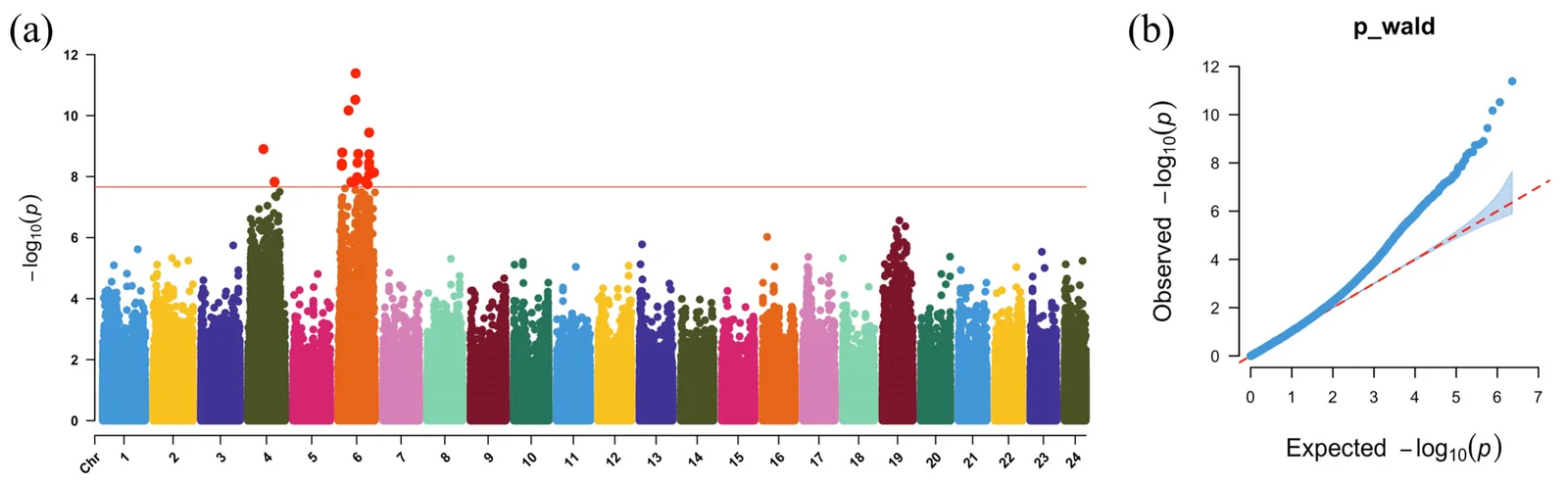
GWAS identified SNPs significantly associated with body weight trait in *S. chuatsi*. (**a**) Manhattan plots for body weight trait. SNPs from different chromosomes are displayed in different colors, with each color representing SNPs located on a different chromosome. Red dots indicate significant SNPs that exceed the Bonferroni-corrected significance threshold. The red line represents the Bonferroni-corrected significance threshold. (**b**) Quantile-quantile plots of GWAS for body weight trait. GWAS (genome-wide association study).

A GWAS of body weight in formulated-diet-adapted mandarin fish was performed using whole-genome resequencing data. Using Bonferroni correction, 21 genome-wide significant SNPs were identified (Figure 2a; Supplementary Table S2). These SNPs were distributed on two chromosomes, with 2 on chromosome 4 and 19 on chromosome 6; chromosome 6 harbored the majority of associated SNPs. The QQ plot showed no apparent deviation from the expected null distribution (Figure 2b). Within ±90 kb of the significant SNPs, 132 protein-coding genes were annotated and considered candidate genes for body weight variation (Supplementary Table S3).

### 2.4. Gene Annotation and Functional Enrichment Analysis of Candidate Genes

Enrichment analysis of the 132 candidate genes identified 87 GO terms and 26 KEGG pathways showing nominal enrichment based on unadjusted *p* values (*p* < 0.05; Supplementary Tables S4 and S5). These nominally enriched terms and pathways could be broadly categorized into two functional themes: lipid metabolic and endocrine pathways, and processes related to neuroendocrine regulation of feeding behavior and energy homeostasis.

#### 2.4.1. Enrichment of Nutrient Utilization and Growth Associated Metabolic Functions and Pathways

**Figure 3:**
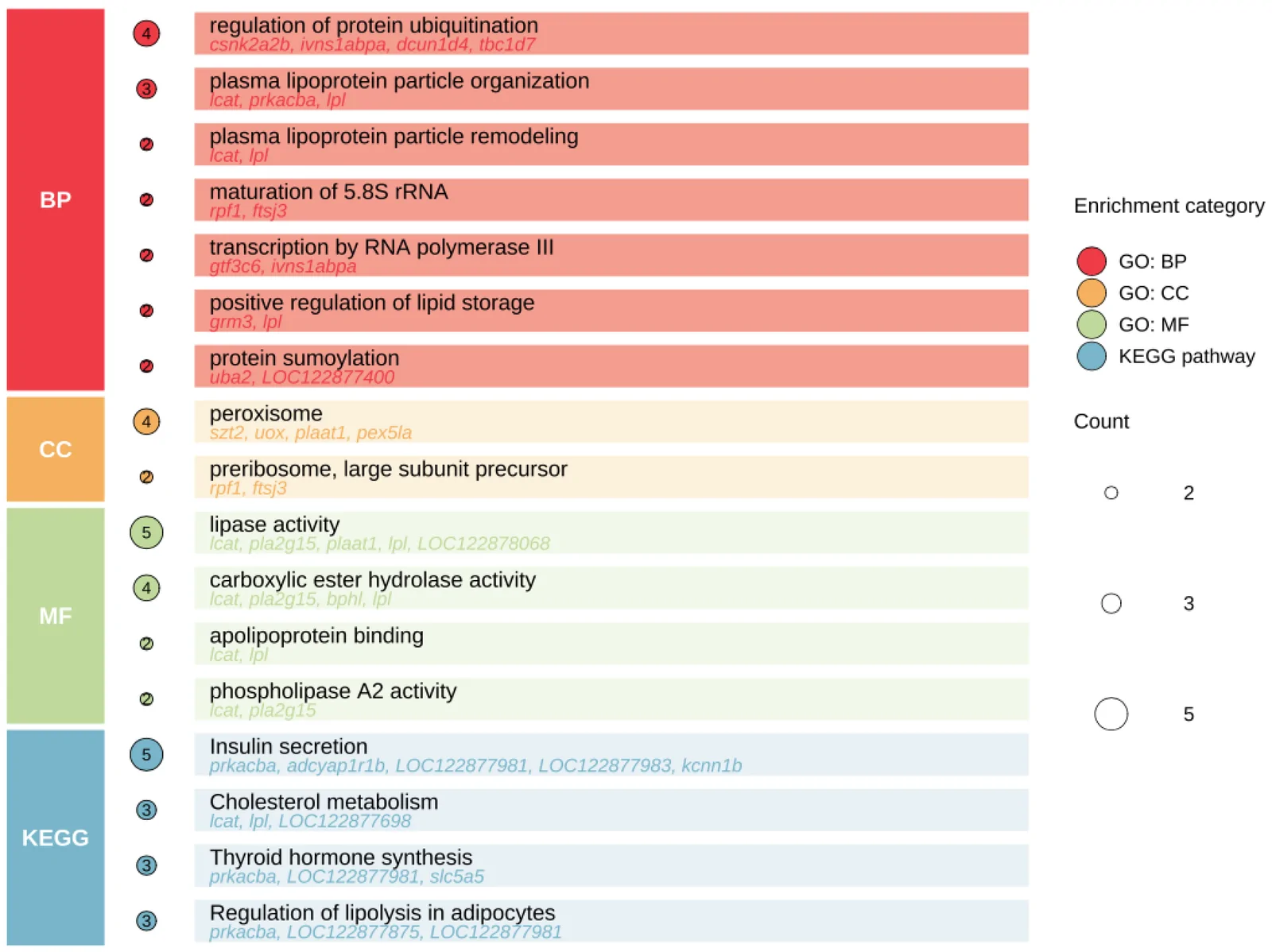
GO and KEGG enrichment analysis of body weight-associated candidate genes related to nutrient utilization and growth-associated metabolic functions. The size of the dots represents the number of enriched genes (Count), and the color of the dots indicates the enrichment category (GO: BP, CC, MF, and KEGG pathway). BP: biological process; CC: cellular component; MF: molecular function; KEGG pathway: Kyoto Encyclopedia of Genes and Genomes pathway.

Enrichment analysis identified GO terms and KEGG pathways associated with cellular processes related to growth, lipid metabolism, and nutrient utilization (Figure 3). Among these, GO terms involved in lipid digestion, transport, and deposition were enriched, including plasma lipoprotein particle organization and remodeling, positive regulation of lipid storage, lipase activity, and phospholipase A_2_ activity. These terms were represented mainly by *LCAT*, *LPL*, *PRKACBA*, *PLA2G15*, and *LOC122877981*. KEGG analysis further identified several metabolic and endocrine-related pathways, including insulin secretion, cholesterol metabolism, thyroid hormone synthesis, and regulation of lipolysis in adipocytes. These findings suggest that body weight-associated candidate genes may contribute to post-feeding nutrient utilization, growth-related metabolic regulation, lipid metabolism, and endocrine metabolic response.

#### 2.4.2. Enrichment of Neuroendocrine Signaling and Feeding-Associated Functions and Pathways

**Figure 4:**
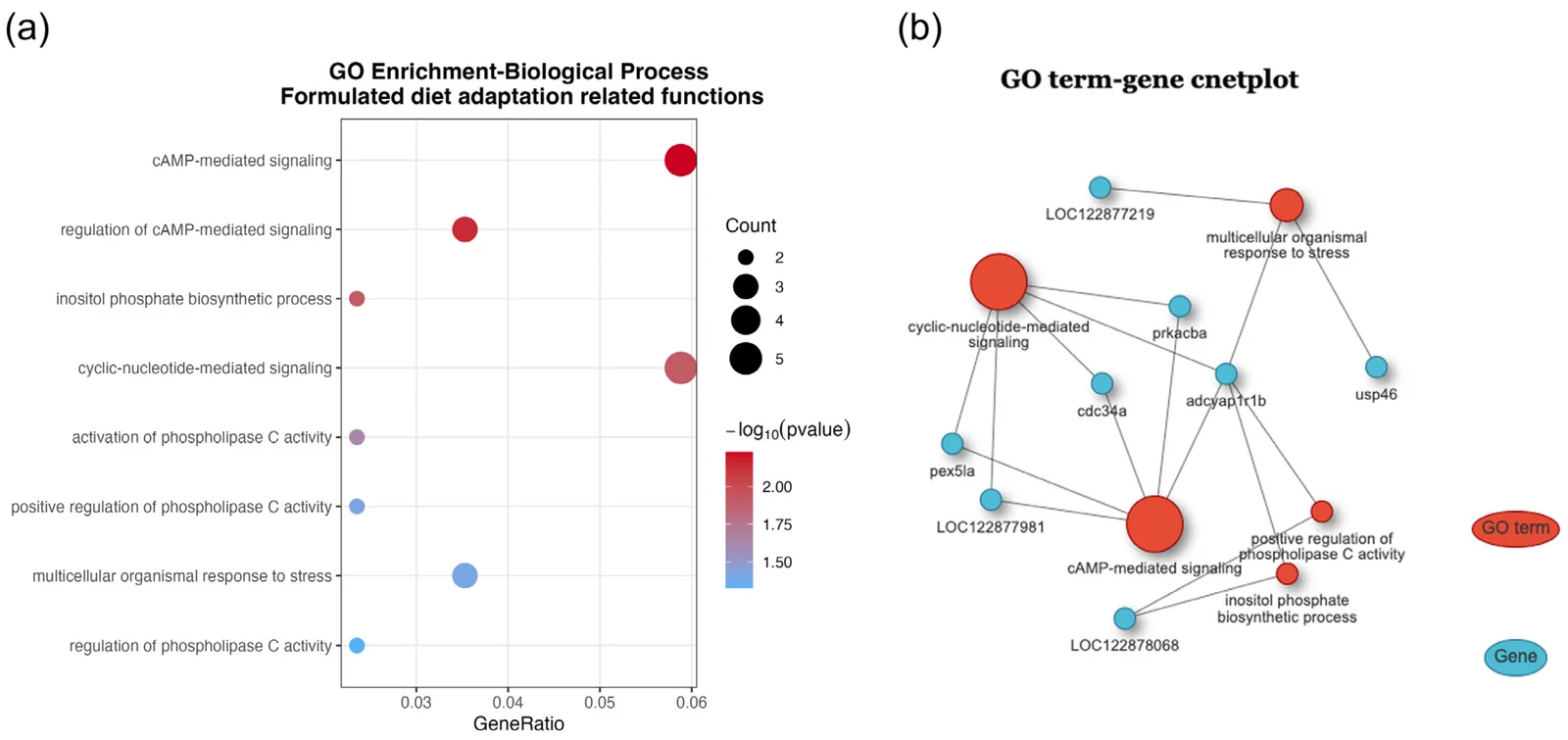
GO enrichment analyses of formulated diet adaptation-related neuroendocrine signaling and feeding-associated functions. (**a**) Dotplot of GO enrichment analysis (*p* < 0.05). The x-axis represents the GeneRatio, the size of the dots represents the number of enriched genes (Count), and the color gradient from blue to red indicates the significance level (−log_10_(*p*-value)). (**b**) Cnetplot of GO terms and genes. The red nodes represent GO terms, the cyan nodes represent genes, and the edges indicate the connections between them.

**Figure 5:**
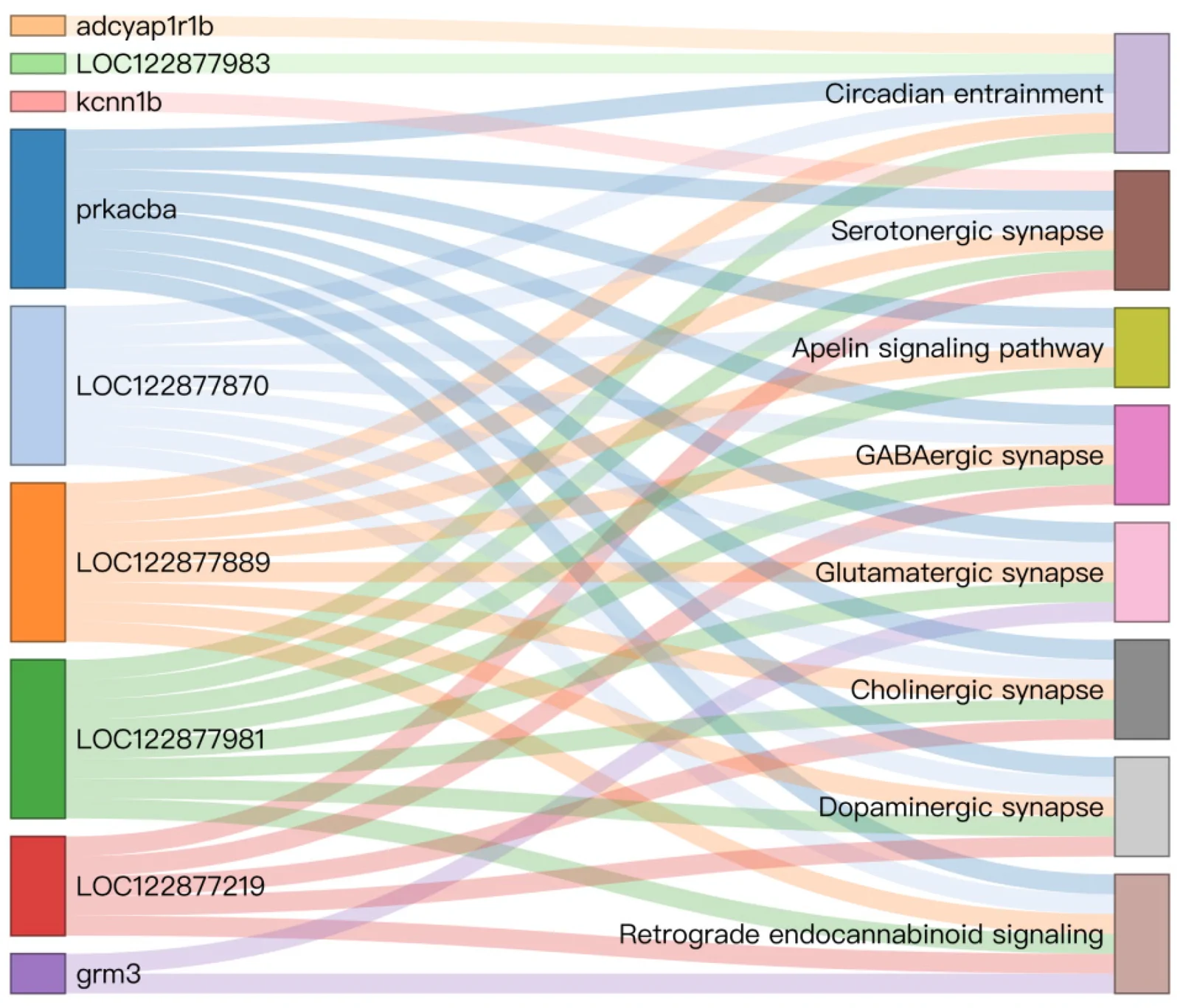
KEGG enrichment analyses of neuroendocrine signaling and feeding-associated pathways.

A second set of nominally enriched functions was associated with neuroendocrine signaling and feeding-related regulation. GO enrichment identified terms including cAMP-mediated signaling, inositol phosphate biosynthetic process, cyclic nucleotide-mediated signaling, positive regulation of phospholipase C activity, and multicellular organismal response to stress (Figure 4a). These terms involved candidate genes such as *ADCYAP1R1B*, *PRKACBA*, *CDC34A*, *PEX5LA*, *LOC122877981*, *LOC122878068*, *LOC122877219*, and *USP46* (Figure 4b). KEGG pathways related to neural signaling and feeding regulation included circadian entrainment, serotonergic synapse, retrograde endocannabinoid signaling, GABAergic synapse, dopaminergic synapse, and the apelin signaling pathway. Several genes, including *PRKACBA*, *LOC122877870*, *LOC122877889*, *LOC122877981*, and *LOC122877219*, were involved in multiple pathways (Figure 5).

### 2.5. Effect of GWAS-Selected Marker Density on Genomic Prediction Performance for Body Weight

**Figure 6:**
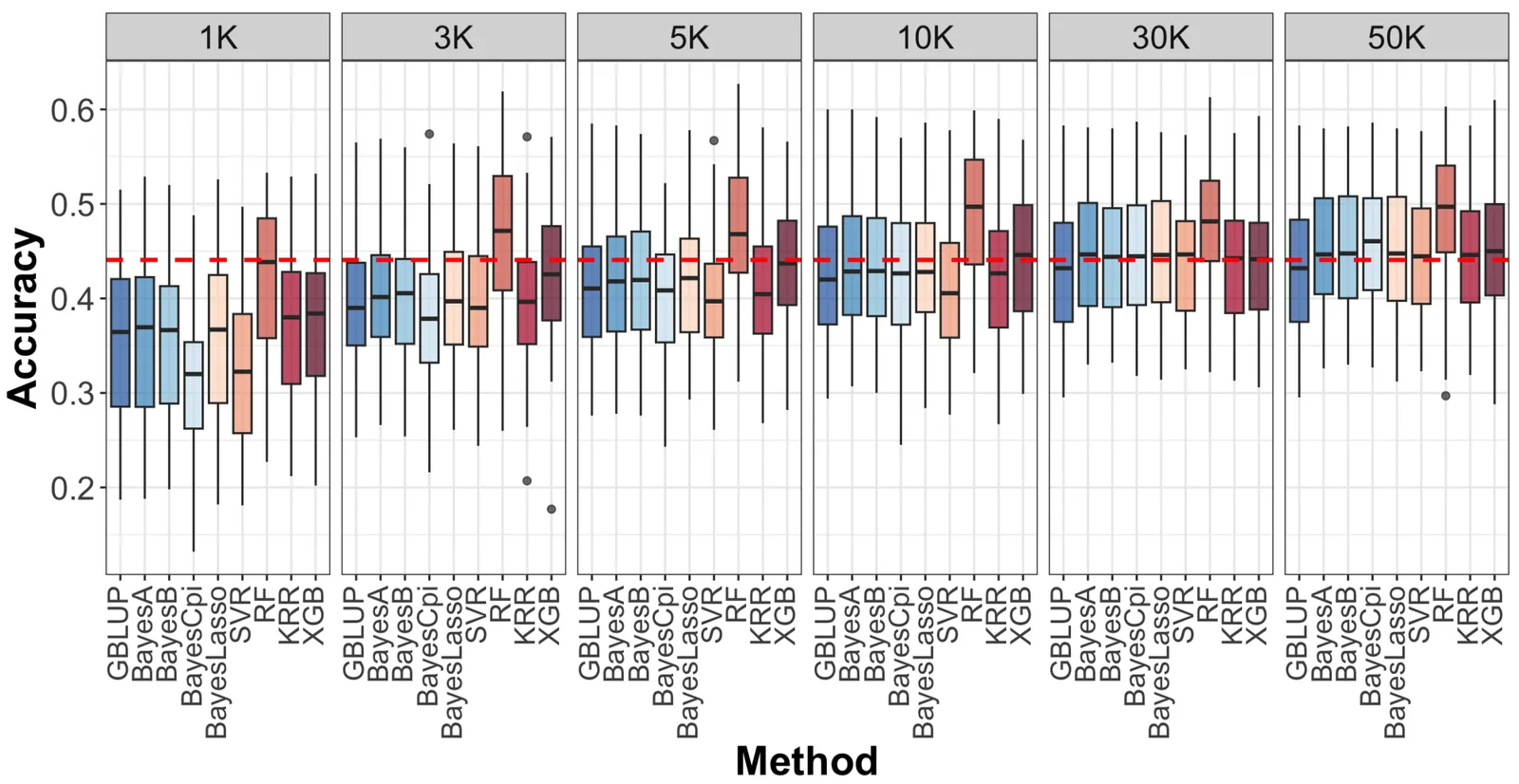
Accuracy of genomic prediction for body weight using GWAS-selected marker sets (1 K–50 K). Linear models included GBLUP, BayesA, BayesB, BayesCπ, and BayesLasso; machine-learning methods included SVR, RF, KRR, and XGB. The red dashed line indicates the prediction accuracy of the WGS-based GBLUP benchmark. Abbreviations: GBLUP, genomic best linear unbiased prediction; GWAS, genome-wide association study; KRR, kernel ridge regression; RF, random forest; SVR, support vector regression; WGS, whole-genome sequencing; XGB, extreme gradient boosting.

Prediction accuracy for body weight depended on both GWAS-selected marker density and prediction model (Figure 6). At 1 K markers, median prediction accuracy for most models was below the whole-genome sequencing (WGS)-based genomic best linear unbiased prediction (GBLUP) benchmark. Accuracy generally increased with marker density. Random forest (RF) was the first model to exceed the benchmark, at 3 K markers, followed by extreme gradient boosting (XGB). At 10 K markers, several models reached or exceeded the benchmark. At 30 K markers, all Bayesian and machine-learning models exceeded the benchmark. At 50 K markers, most models performed comparably to or better than the benchmark. Across all marker densities, RF achieved the highest prediction accuracy among the evaluated methods.

**Figure 7:**
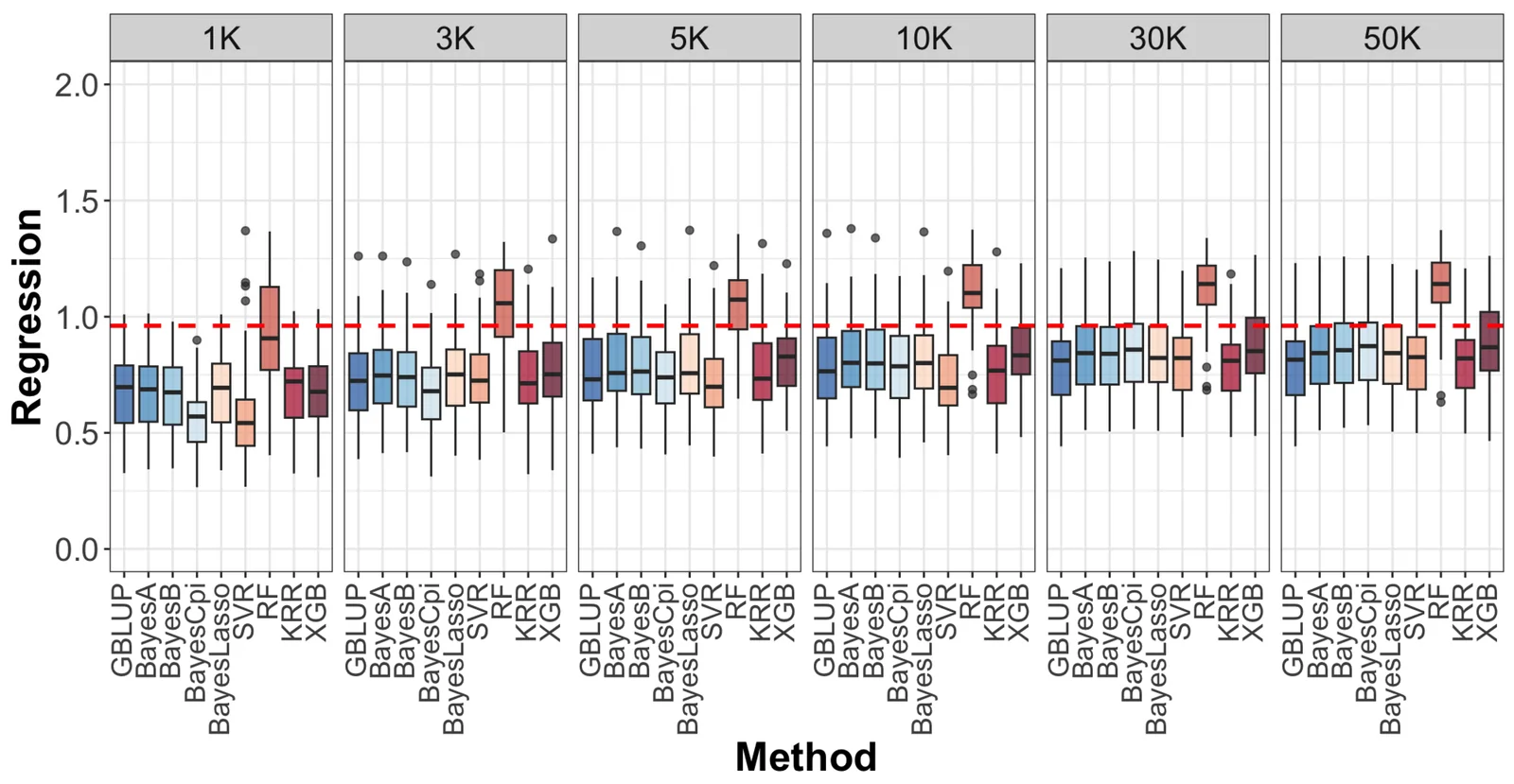
Regression coefficients (calibration slopes) of genomic prediction for body weight using GWAS-selected marker sets (1 K–50 K markers). Observed body weight was regressed on predicted genomic estimated breeding values (GEBVs). The red dashed line indicates the WGS-based GBLUP benchmark. Models and abbreviations are as in Figure 6.

The calibration slopes of genomic prediction for body weight are shown in Figure 7. At 1 K markers, most models had median slopes below the WGS-based GBLUP benchmark. With increasing marker density, the slopes generally increased and approached the benchmark. RF reached or exceeded the benchmark at 3 K markers, followed by extreme gradient boosting (XGB) and kernel ridge regression (KRR) at higher marker densities. At 30 K and 50 K markers, RF, KRR, and XGB had median slopes comparable to or slightly above the benchmark, whereas GBLUP and the Bayesian models remained below the benchmark. Among all methods, RF showed the highest median calibration slope across marker densities, although its slope distribution was wider and included several outliers. Overall, calibration slopes increased with GWAS-selected marker density, and RF consistently showed the highest median slope across marker densities.

## 3. Discussion

### 3.1. Genetic Architecture Underlying Body Weight Variation Following Formulated Diet Adaptation

We characterized the genetic architecture of body weight variation in formulated-diet-adapted *S. chuatsi*. Despite uniform diet adaptation and subsequent pond rearing, body weight varied widely among individuals, and the estimated narrow-sense genomic heritability was 0.684 ± 0.081, indicating a substantial genetic contribution. GWAS detected genome-wide significant associations concentrated on chromosome 6, with a smaller number on chromosome 4, consistent with polygenic control and a prominent hotspot on chromosome 6. Functional annotation of the candidate genes implicated nutrient utilization and growth-associated metabolism, as well as neuroendocrine regulation of appetite and energy homeostasis. Previous studies on formulated-diet adaptation in mandarin fish have mainly focused on diet acceptance and epigenetic or expression changes [8,12,13,20,21]. The present study extends these findings by linking genomic variation to growth performance after successful adaptation.

#### 3.1.1. Lipid Metabolic and Endocrine Pathways Underlying Body Weight Variation

The present GWAS identified candidate genes and enriched pathways associated with body weight variation in formulated-diet-adapted mandarin fish, with prominent signals related to lipid metabolism and endocrine regulation. The co-occurrence of *LPL*, *LCAT*, and *PLA2G15* suggests that growth differences may involve variation in lipid acquisition, transport, remodeling, and intracellular utilization after adaptation to formulated diets.

Among these candidates, *LPL* is of particular interest. As a key enzyme that hydrolyzes triglyceride-rich lipoproteins and facilitates fatty acid uptake by peripheral tissues, *LPL* regulates lipid availability for energy metabolism and tissue growth [22]. In mandarin fish, *LPL* polymorphisms, methylation patterns, and hepatic expression differences have been associated with formulated-diet acceptance [8,20,22]. The *LPL* variants associated with body weight here extend these findings by suggesting that lipid utilization may influence not only the capacity to accept formulated diets but also subsequent growth performance among adapted individuals. In addition to *LPL*, *LCAT* and *PLA2G15* were identified, indicating that multiple stages of lipid metabolism may contribute to growth variation. *LCAT* participates in cholesterol esterification and lipoprotein remodeling, whereas *PLA2G15* is involved in lysosomal phospholipid turnover and intracellular lipid homeostasis [23,24]. Although direct evidence linking these genes to fish growth traits remains limited, their functions, together with those of *LPL*, suggest that coordinated regulation of lipid processing, rather than a single metabolic pathway, may underlie body weight differences under formulated-diet conditions.

Beyond lipid metabolism, enrichment of insulin secretion, thyroid hormone synthesis, and lipolysis-related pathways points to a possible role of endocrine regulation in metabolic variation. In particular, *PRKACBA* and *LOC122877981* may participate in cAMP-mediated signaling, which integrates hormonal and nutritional cues to regulate downstream metabolic processes. Previous epigenetic studies have also implicated cAMP-related regulation, including altered methylation of *PRKACBA*, in mandarin fish differing in formulated-diet acceptance [8]. Although the present GWAS associations do not establish causal effects and require functional validation, they support a genetic framework in which lipid metabolic capacity and endocrine regulation jointly contribute to body weight variation in formulated-diet-adapted mandarin fish. In contrast to previous studies that focused primarily on the genetic basis of formulated-diet acceptance, the present findings suggest that variation in metabolic efficiency after successful adaptation may be an important determinant of growth performance.

#### 3.1.2. Neuroendocrine Regulation of Feeding Behavior and Energy Homeostasis

Beyond lipid metabolism, the candidate genes identified in the present study were enriched in neuroendocrine-related processes, including cAMP signaling, regulation of phospholipase activity, stress responses, and multiple neurotransmitter synaptic pathways. Several genes, including *ADCYAP1R1B*, *PRKACBA*, *CDC34A*, *PEX5LA*, *LOC122877981*, and *LOC122878068*, contributed to these enriched categories, suggesting that variation in neuroendocrine regulation may represent another mechanism underlying body weight differences among formulated-diet-adapted individuals.

Among these genes, *ADCYAP1R1B* showed particularly strong functional relevance, as it was repeatedly detected across multiple enriched GO categories. This gene encodes a receptor for pituitary adenylate cyclase-activating polypeptide (PACAP), a neuropeptide involved in the regulation of feeding-related endocrine responses in teleosts [25]. Previous studies have suggested that PACAP signaling can influence appetite regulation through interactions with hypothalamic neuroendocrine pathways, including corticotropin-releasing hormone (CRH)-mediated signaling [25,26,27]. In mandarin fish, previous genomic studies of feeding habit domestication mainly identified selection signals and regulatory changes associated with the transition from live prey to formulated diets, particularly involving sensory perception, neural function, and metabolic regulation [8,20]. In contrast, the present study identified variation in *ADCYAP1R1B* and related neuroendocrine genes among individuals that had already acquired formulated diet acceptance, suggesting that these pathways may contribute not to the initial establishment of feeding behavior but to differences in feeding regulation, physiological state, and growth potential after adaptation.

The enrichment of stress-related biological processes further indicates that neuroendocrine regulation may influence growth indirectly by affecting feeding stability and energy allocation. Previous studies have shown that formulated diet domestication can induce changes in oxidative stress responses and antioxidant capacity in mandarin fish [28]. Therefore, genetic variation affecting stress responsiveness may influence the ability of individuals to maintain stable feeding activity and allocate energy toward growth rather than physiological stress responses.

KEGG enrichment analysis further revealed enrichment of multiple neurotransmission-related pathways, including serotonergic, GABAergic, glutamatergic, cholinergic, and dopaminergic synapses, as well as the apelin signaling pathway. These pathways are known to participate in the neural regulation of feeding and energy homeostasis. Previous studies have shown that serotonergic, GABAergic, and endocannabinoid systems contribute to central control of food intake [29], while dopaminergic circuits can modulate feeding motivation and satiety [30]. In teleosts, apelin signaling has also been implicated in appetite and growth regulation, with Pyr-apelin-13 altering the expression of appetite- and growth-related genes in common carp [31] and apelin functioning as an orexigenic factor in goldfish [32]. Similarly, the endocannabinoid system has been shown to promote food intake in rainbow trout and goldfish [33,34]. In the present study, several candidate genes, including *PRKACBA*, *LOC122877870*, *LOC122877889*, *LOC122877981*, and *LOC122877219*, were involved in or shared across these feeding-related pathways, suggesting that body weight variation among formulated-diet-adapted mandarin fish may be influenced by coordinated regulation of neuroendocrine networks controlling appetite, feeding behavior, and energy allocation. These findings extend previous studies on formulated-diet domestication by indicating that neuroendocrine pathways may continue to contribute to phenotypic variation after feeding adaptation has been established. Whereas earlier studies focused primarily on the genetic basis of whether mandarin fish can accept formulated diets, the present results suggest that individual differences in appetite-related regulation, stress responsiveness, and energy homeostasis may further influence growth performance under formulated-diet conditions. Functional validation of these candidate genes will be necessary to clarify their roles in feeding efficiency and body weight variation.

### 3.2. Effects of SNP Density on Genomic Prediction

Prediction accuracy increased with SNP density under GWAS-informed marker selection, with limited improvement beyond approximately 30 K SNPs. Similar trends have been reported in other aquaculture species, where GWAS-informed SNP subsets achieved prediction accuracy comparable to or higher than that of full WGS marker sets for growth and production traits in sturgeon, Atlantic salmon, common carp, and rainbow trout [35,36]. At this density and above, GWAS-prioritized panels consistently achieved high prediction accuracy for body weight, indicating that a moderate-density panel may be sufficient for genomic prediction in this population. Genomic relationship matrix analyses supported this pattern: the correlation between the WGS-based G matrix and SNP subset-derived matrices increased from 0.74 at 1 K SNPs to 0.88 at 50 K SNPs, whereas matrices based on 10 K–50 K SNPs were highly correlated (r = 0.99–1.00), indicating that genomic relationship estimates stabilized at moderate-to-high marker densities (Supplementary Figure S1).

Increasing marker number alone did not necessarily improve prediction. Although WGS provides broad genome coverage, some variants may contribute limited additional information because of redundancy or weak associations with the target trait. Low- or medium-density panels combined with imputation can maintain high prediction accuracy when training and validation populations are closely related [37,38]. In the present population, a panel of approximately 30 K informative SNPs therefore appears to balance prediction accuracy and genotyping efficiency for body weight improvement in formulated-diet-adapted mandarin fish. Reducing marker density while preserving predictive performance would make genomic selection more cost-effective in future breeding programs.

### 3.3. Comparison of Linear and Machine Learning Models on Genomic Prediction

Genomic prediction performance for body weight in formulated-diet-adapted mandarin fish depended on both SNP density and model choice. Among linear models, BayesA, BayesB, and BayesLASSO generally yielded slightly higher prediction accuracies than GBLUP, whereas BayesCπ showed lower accuracy at low SNP densities but improved as marker density increased, reaching the highest median accuracy among Bayesian models at 50 K SNPs. Overall, differences among Bayesian models and GBLUP were modest, consistent with the polygenic architecture of body weight, in which many loci of small effect collectively contribute to phenotypic variation.

Machine-learning models differed more markedly in performance. Random forest (RF) consistently achieved the highest or near-highest accuracy across SNP densities and outperformed GBLUP, Bayesian models, Support vector regression (SVR), KRR, and XGB in most scenarios. This advantage may reflect the ability of RF to capture nonlinear relationships and interactions among markers without predefined assumptions about marker effects [39]. However, RF also showed regression coefficients above the reference value, indicating potential prediction bias despite its superior accuracy. Both accuracy and calibration therefore need to be considered when evaluating genomic prediction models.

Model performance also varies with trait architecture, species, and population characteristics. In sturgeon, prioritized SNP selection from whole-genome sequencing improved prediction accuracy, and machine-learning models performed competitively relative to conventional linear approaches [36], supporting the present finding that biologically informed marker selection combined with suitable prediction models can enhance genomic prediction efficiency. In Holstein heifers, machine-learning and conventional models using low-coverage sequencing data achieved comparable performance for fertility traits, highlighting the potential of cost-effective strategies [35]. In rainbow trout, BayesC achieved relatively high accuracy for resistance to *Piscirickettsia salmonis*, and a limited number of SNPs sufficed to match higher-density panels [40]. In eastern oyster, BayesB outperformed Bayesian ridge regression and RKHS for low-salinity tolerance, suggesting that models allowing heterogeneous marker effects are advantageous when loci have unequal effects [41]. These findings are consistent with the present results, where Bayesian models performed stably but their relative advantage depended on SNP density.

Machine-learning approaches have also shown promise for complex aquaculture traits. In olive flounder (*Paralichthys olivaceus*), RF, Elastic Net, and BayesB achieved higher accuracies than GBLUP for growth traits [42], supporting the superior performance of RF observed here. Similarly, artificial intelligence-based algorithms improved genomic prediction for disease resistance in striped catfish compared with conventional methods [43], and machine-learning models showed advantages for growth-trait prediction in Pacific white shrimp [44]. However, the optimal model differed across studies, indicating that prediction performance depends on the interaction between model assumptions, genetic architecture, marker density, and population structure. Combining GWAS-informed SNP selection with an appropriate prediction model therefore provides an efficient strategy for genomic selection of body weight in formulated-diet-adapted mandarin fish. Among the approaches evaluated, RF showed the highest predictive potential, whereas Bayesian models offered relatively stable performance across SNP densities. These results provide a practical basis for optimizing genomic prediction and reducing genotyping costs in future breeding programs.

### 3.4. Influence of GWAS-Based SNP Prioritization on Genomic Prediction Accuracy

The present findings indicate that GWAS-based SNP prioritization can improve genomic prediction accuracy in this population, consistent with previous studies. In sturgeon, GWAS-prioritized SNPs increased accuracy by 3.4% over the full WGS dataset at moderate marker densities (≥10 K) [36]. In *Siniperca scherzeri*, 500 GWAS-selected SNPs achieved a prediction ability of 0.70 for body length and total length [45]. A GBLUP variant incorporating BLINK-derived GWAS information (GAGBLUP) improved accuracy by more than 20% over conventional GBLUP using all genome-wide markers [46]. These results support GWAS-assisted marker selection as an efficient strategy for genomic prediction in specific populations and traits.

Model evaluation should also consider error-based metrics. In the present study, GBLUP consistently showed the highest median mean squared error (MSE) and mean absolute error (MAE) across SNP densities, whereas the evaluated Bayesian and machine-learning models had substantially lower and comparable error distributions (Supplementary Figures S2 and S3). This indicates that, although GBLUP served as a useful benchmark, Bayesian and machine-learning approaches achieved lower prediction errors for body weight in this population. Because RF showed high accuracy but also regression bias, model selection should jointly consider accuracy, calibration, and error-based metrics.

### 3.5. Limitations and Future Perspectives

Several limitations should be considered when interpreting these findings. Although GWAS identified significant SNPs and candidate genes associated with body weight variation, their biological functions and potential regulatory mechanisms remain to be experimentally validated. Future studies should focus on functional characterization of these candidate genes to better understand the molecular mechanisms underlying growth variation in formulated-diet-adapted mandarin fish. Furthermore, multi-generational selection experiments are needed to evaluate the long-term effectiveness and practical applicability of GWAS-informed SNP panels in breeding programs. Integrating genomic information with transcriptomic, epigenomic, or metabolomic data may further improve our understanding of growth regulation and enhance genomic prediction accuracy.

## 4. Materials and Methods

### 4.1. Population and Phenotype

All animal procedures complied with animal care laws and guidelines and were approved by the Ethics Committee of the Beijing Academy of Agriculture and Forestry Sciences (BAAFS-KJLL-20250509; 9 May 2025). Healthy juvenile all-female *S. chuatsi* were obtained from Guangdong Liangshi Aquatic Seed Industry Co., Ltd. (Foshan, China) and transported to the Sanshui Research and Experimental Base of the Pearl River Fisheries Research Institute, Chinese Academy of Fishery Sciences (Foshan, Guangdong, China). Fish were initially fed live fry of mud carp (*Cirrhinus molitorella*). At approximately 4 cm body length, they underwent a 25-day gradual transition from live prey to a formulated diet, following the procedure described previously [47]. During adaptation, individuals that fed actively, swam vigorously, and had well-filled abdominal cavities were retained, whereas those with poor feed acceptance or low vitality were removed. Representative images of individuals with high and low feed-adaptation performance are shown in Supplementary Figure S4. The retained individuals constituted the formulated-diet-adapted population.

The adapted fish were subsequently reared in a single earthen pond (~0.67 ha) on the same formulated diet until 1 year of age. Water quality was monitored daily. Temperature, pH, and dissolved oxygen were measured using a YSI multiparameter water quality meter (YSI Inc., Yellow Springs, OH, USA), and ammonia nitrogen and nitrite nitrogen were determined using commercial test kits according to the manufacturer’s instructions. Over the one-year rearing period, water quality was maintained as follows: temperature averaged 26.3 ± 6.5 °C (range, 20–32 °C), dissolved oxygen > 6 mg/L, pH averaged 7.9 ± 0.23, ammonia nitrogen < 0.1 mg/L, and nitrite nitrogen < 0.05 mg/L. Water exchanges were performed with temperature-equilibrated water. No obvious clinical signs or abnormal behaviors were observed during the rearing period.

A total of 522 one-year-old fish were randomly selected for phenotypic measurement and genomic analysis. Fish were anesthetized with eugenol, weighed using an electronic balance (±0.01 g), and caudal fin tissues were collected and preserved in 95% ethanol for whole-genome resequencing. Descriptive statistics for body weight (BW), including minimum, maximum, mean, standard deviation (SD), and coefficient of variation (CV), were calculated.

### 4.2. Whole-Genome Resequencing and Variant Calling

Genomic DNA was extracted from caudal fin tissue using the DNeasy Blood & Tissue Kit (Qiagen, Hilden, Germany) [48]. DNA concentration and integrity were assessed using a NanoDrop microvolume spectrophotometer (Thermo Fisher Scientific, Waltham, MA, USA) and 1.0% agarose gel electrophoresis, respectively. Paired-end libraries with 150-bp reads were prepared and sequenced on the DNBSEQ T7 platform (MGI Tech Co., Ltd., Shenzhen, China) by BGI Genomics Co., Ltd. (Shenzhen, China), with an expected average sequencing depth of approximately 20× per individual.

Raw reads were processed using fastp (v0.23.2) [49] to remove adapter sequences, low-quality bases, and reads containing >10% N bases. Clean reads were aligned to the *Siniperca chuatsi* reference genome (assembly ASM2008510v1) using BWA-MEM (v0.7.17) [50]. Aligned reads were sorted using SAMtools (v1.19) [51], and PCR duplicates were removed using sambamba (v1.0.1) [52]. Variants were called using GATK HaplotypeCaller (v4.1.3.0) following the best-practice workflow, and the resulting gVCF files were combined using CombineGVCFs. Variant quality filtering was performed using VariantFiltration with the following criteria: QUAL < 30.0, QD < 2.0, FS > 60.0, MQ < 40.0, MQRankSum < −12.5, and ReadPosRankSum < −8.0. Missing genotypes were imputed using Beagle 4.1 [53]. Final SNP quality control was performed using PLINK (v1.90) [54], retaining biallelic SNPs with a call rate >90%, minor allele frequency >0.01, and Hardy–Weinberg equilibrium *p* > 1 × 10^−6^.

### 4.3. Population Structure and Linkage Disequilibrium Analysis

Population structure was evaluated by PCA based on the genomic relationship matrix constructed from genome-wide SNPs using GCTA (v1.25.3) [55]. The first three principal components were used to visualize genetic relationships among individuals. To control for potential population stratification in the GWAS, the first 5 to 10 principal components were evaluated as covariates, and the corresponding genomic inflation factor (λ) was calculated for each model (Supplementary Table S1). A λ value closer to 1 indicates better control of potential inflation of association statistics and was considered when selecting the optimal number of PCs [56].

Genome-wide linkage disequilibrium was quantified using the squared correlation coefficient (*r*^2^) with PopLDdecay (v3.42) [57]. The physical distance corresponding to the genome-wide average r^2^ was used to define the LD decay distance and the candidate-gene search window.

### 4.4. Genome-Wide Association Analysis

Genome-wide association analysis was performed using the linear mixed model (LMM) implemented in GEMMA (v0.98.5) [58] to identify single-nucleotide polymorphisms (SNPs) associated with body weight. A genetic relationship matrix (GRM) was constructed from all SNPs to account for genetic relatedness among individuals. The narrow-sense genomic heritability (*h*^2^) of body weight was estimated using a GBLUP model based on the GRM as *h*^2^ = $\frac{\sigma_{g}^{2}}{\sigma_{g}^{2}\;+\;\sigma_{e}^{2}}$, where $\sigma_{g}^{2}\;$and $\sigma_{g}^{2}$ represent additive genetic and residual variances, respectively. All three tests (Wald, likelihood ratio, and score) were performed using the -lmm 4 option. Genome-wide significance was determined using the Bonferroni-corrected threshold (*p* < 0.05/N), where N is the number of SNPs tested. Manhattan and quantile–quantile (QQ) plots were generated using the CMplot package [59] in R. Based on the LD decay distance determined in Section 4.3, candidate genes were annotated within ±90 kb of significant SNPs. For genomic prediction, GWAS-based SNP selection was performed independently within the training population of each cross-validation fold, excluding the corresponding validation population from GWAS and SNP prioritization, to prevent information leakage. The selected SNP panels were then used for prediction in the corresponding validation population.

### 4.5. Gene Annotation and Functional Enrichment Analysis

Protein-coding genes of *S. chuatsi* (assembly ASM2008510v1) were functionally annotated using eggNOG-mapper v2.1.15 [60]. Candidate genes identified by GWAS were subjected to Gene Ontology (GO) and Kyoto Encyclopedia of Genes and Genomes (KEGG) enrichment analyses using the clusterProfiler R package (v4.3.3) [61]. Multiple-testing correction was performed using the Benjamini–Hochberg (BH) method [62]. Enriched terms and pathways were evaluated based on both nominal *p* values and BH-adjusted *p* values, with *p* < 0.05 and adjusted *p* < 0.05 considered nominal and statistically significant enrichment thresholds, respectively.

### 4.6. GWAS-Informed SNP Panel Construction

To determine whether GWAS-informed marker reduction could preserve predictive information while decreasing marker density, SNPs were ranked by ascending GWAS *p*-values. Six panels containing the top 1 K, 3 K, 5 K, 10 K, 30 K, and 50 K SNPs were then constructed [36]. These reduced panels were compared with the full genome-wide SNP set to assess the effect of marker density on genomic prediction. Because the full SNP set was computationally prohibitive for Bayesian and machine-learning models, GBLUP was used as the benchmark for the full SNP set, whereas the six GWAS-informed panels were evaluated using GBLUP [63], Bayesian models, and machine-learning algorithms.

### 4.7. Genomic Prediction Models

Genomic prediction of BW was performed using GBLUP, four Bayesian alphabet models (BayesA and BayesB [64], BayesCπ [65], and Bayesian LASSO [66]), and four machine-learning algorithms: SVR [67], RF [68], KRR [69], and XGBoost [70].

GBLUP was implemented in DMU v6 [71]. Genomic relationship matrices were constructed from the full WGS-derived SNP set and from training-fold-specific GWAS-selected SNP panels. Variance components were estimated by restricted maximum likelihood using the average information algorithm, and GEBVs were obtained using the DMUAI procedure.

Bayesian models were implemented using the BGLR R package v1.1.4 [72]. These models differ in their assumptions regarding the distribution and magnitude of SNP effects, allowing evaluation of alternative genetic architectures. For each Bayesian model, 18,000 Markov chain Monte Carlo (MCMC) iterations were run, with the first 3000 discarded as burn-in. GEBVs were calculated as the posterior means of genetic values from the retained iterations.

SVR, RF, KRR, and XGBoost were implemented in Python v3.8.3 using scikit-learn v1.3.0 [73,74,75] and XGBoost v1.7.6 [70]. Hyperparameters were optimized by grid search with an inner cross-validation loop within each training fold, using the same training-fold-specific marker panel. The validation fold was not used for marker selection, hyperparameter tuning, or model training. Because the full WGS marker set was computationally demanding for Bayesian and machine-learning models, these models were evaluated using the six GWAS-selected marker densities (1 K, 3 K, 5 K, 10 K, 30 K, and 50 K). GBLUP was additionally evaluated using the full WGS-derived SNP set as the benchmark.

### 4.8. Benchmarking of Genomic Prediction Accuracy

All models were evaluated using repeated five-fold cross-validation [76]. The 522 individuals were randomly divided into five approximately equal-sized folds; four folds were used for training and the remaining fold for validation. Each fold served once as the validation set. The entire five-fold procedure was repeated 20 times using independent random partitions [77]. Within each repetition, identical fold assignments were used across all models and marker densities to ensure a fair comparison, and random seeds were fixed to ensure reproducibility. Marker panels were constructed as described in Section 4.6. In brief, GWAS was performed independently within the training population of each cross-validation fold, and the top SNPs were used to construct the 1 K, 3 K, 5 K, 10 K, 30 K, and 50 K panels; the validation population was excluded from GWAS and SNP prioritization. Predictions for each validation fold were generated using the model and marker panel trained on the corresponding training folds.

Prediction performance was assessed using the Pearson correlation coefficient between observed BW and predicted GEBVs [78]. Prediction bias was assessed by regressing observed BW on predicted GEBVs; the regression slope (calibration slope) was reported, with a slope of 1 indicating no systematic bias. MSE and MAE were additionally calculated to assess prediction error and overall model performance [77].

## 5. Conclusions

This study represents the first application of genomic selection for body weight prediction in formulated-diet-adapted *S. chuatsi*. Body weight exhibited substantial phenotypic variation and high genomic heritability, indicating considerable potential for genetic improvement through selection. By integrating GWAS with genomic prediction, we demonstrated that GWAS-prioritized SNP panels, particularly those containing approximately 30 K markers, achieved prediction performance comparable to or better than the whole-genome marker set, with random forest showing the highest accuracy among evaluated models. The identified candidate genes and pathways provide insights into the genetic basis of body weight variation after formulated-diet adaptation and support the application of GWAS-informed genomic selection in aquaculture breeding.

## Figures and Tables

**Table 1:** Descriptive statistics and heritability estimate for body weight.

Category	Statistic	Value
Population	Number of fish	522
Body weight (g)	Mean ± SD	487.75 ± 277.59
	CV (%)	56.91
	Minimum	55
	Maximum	1461.2
Genetic parameter	Heritability (*h*^2^) ± SE	0.684 ± 0.081

Note: SD means standard deviation; CV means coefficient of variation; SE means standard error.

## Data Availability

The datasets used during the current study are available from the corresponding author on reasonable request.

## Supplementary Materials

The following supporting information can be downloaded at: https://www.mdpi.com/article/10.3390/ijms27219433/s1.

## Abbreviations

The following abbreviations are used in this manuscript:

GBLUP	Genomic best linear unbiased prediction
GEBV	Genomic estimated breeding value
GRM	Genetic relationship matrix
GS	Genomic selection
GWAS	Genome-wide association study
KRR	Kernel ridge regression
LD	Linkage disequilibrium
MAE	Mean absolute error
MSE	Mean squared error
PCA	Principal component analysis
r^2^	Squared correlation coefficient
RF	Random forest
SNP	Single-nucleotide polymorphism
SVR	Support vector regression
WGS	Whole-genome sequencing/resequencing
XGBoost	Extreme gradient boosting
λ	Genomic inflation factor
