# Supplementary material for: Genetic Architecture of Body Weight Variation and GWAS-Informed Genomic Selection in Formulated Diet-Adapted Mandarin Fish (Siniperca chuatsi)
Source: Int J Mol Sci. 2026 Oct 22;27(21):9433. doi: 10.3390/ijms27219433 (PMC13762688; doi:10.3390/ijms27219433)
Supplement: Supplementary file 1 [file ijms-27-09433-s001.zip › ijms-4591420-supplementary/Supplementary Files/Supplementary_figures-261012.pdf]

Supplementary Figures

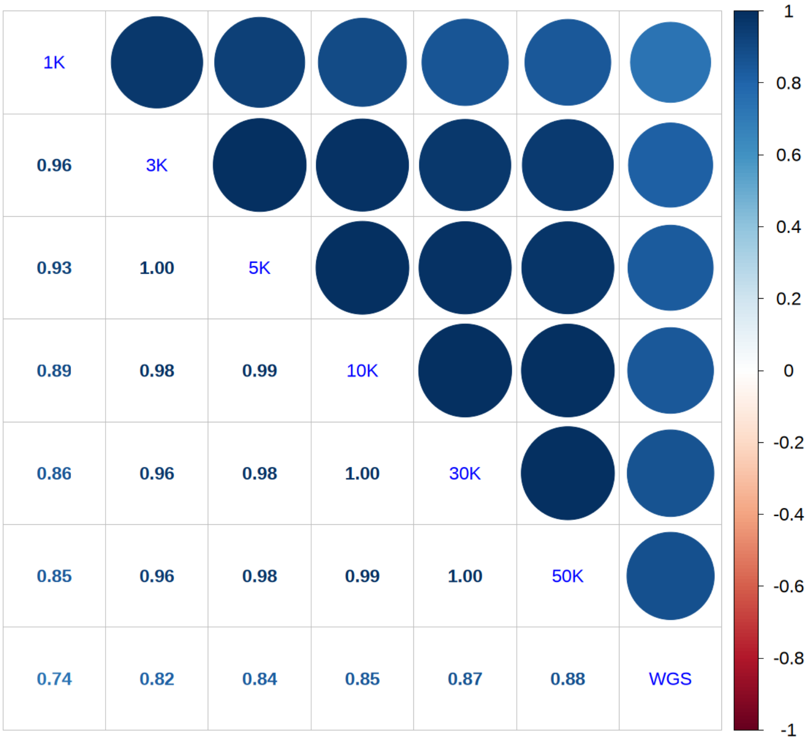

Figure S1. Pairwise correlations among genomic relationship matrices (G matrices) constructed using SNP panels of different densities and the full whole-genome sequencing (WGS) dataset. Numerical values in the lower triangle represent the correlation coefficients between pairs of G matrices, while circle size and color intensity in the upper triangle indicate the magnitude of the correlations.

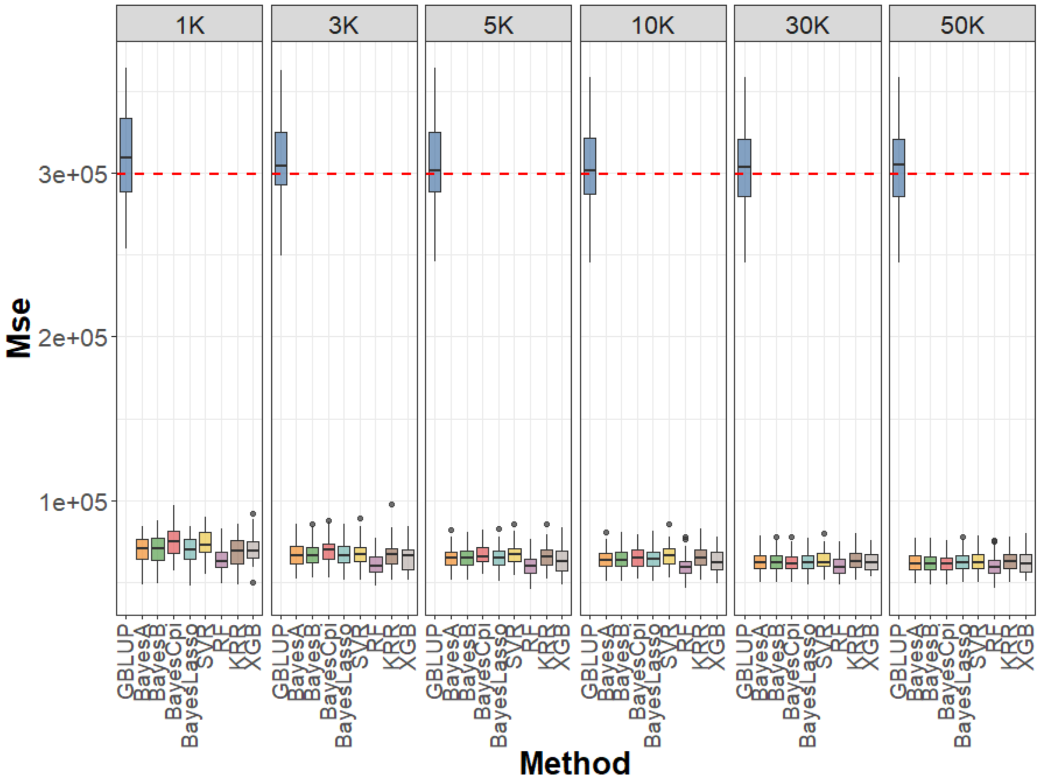

Figure S2. Mean squared error (Mse) of genomic prediction for body weight based on GWAS across different SNP densities, using linear and machine learning models.

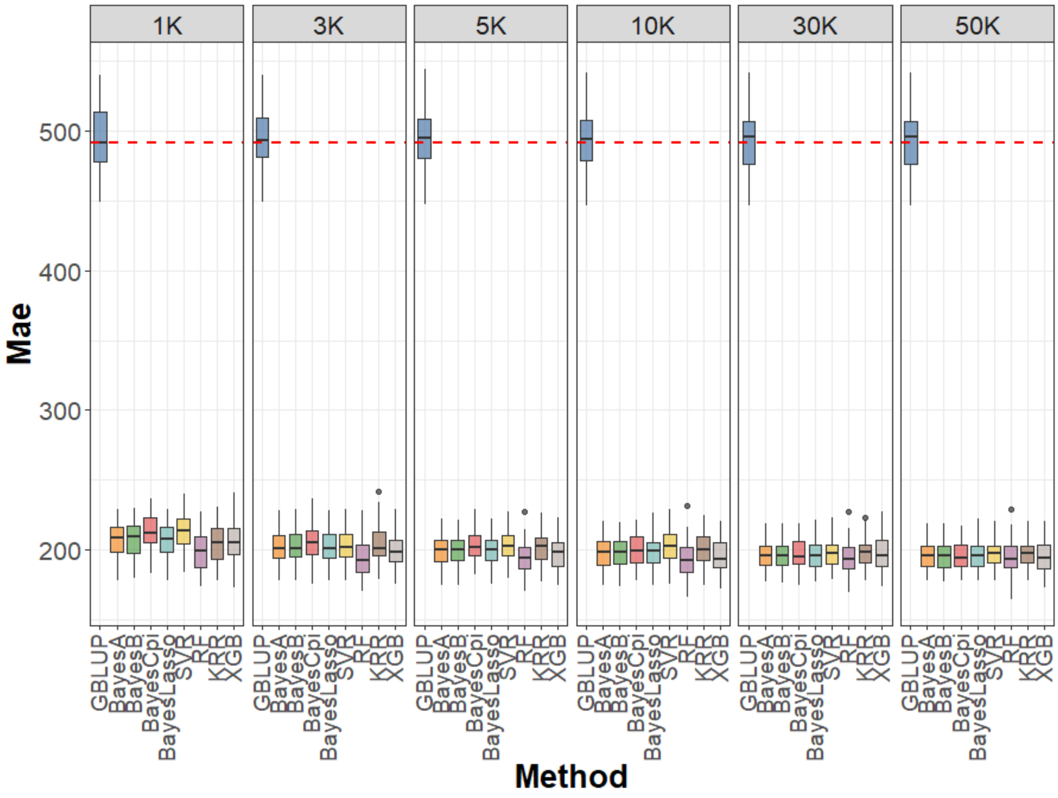

Figure S3. Mean absolute error (Mae) of genomic prediction for body weight based on GWAS across different SNP densities, using linear and machine learning models.

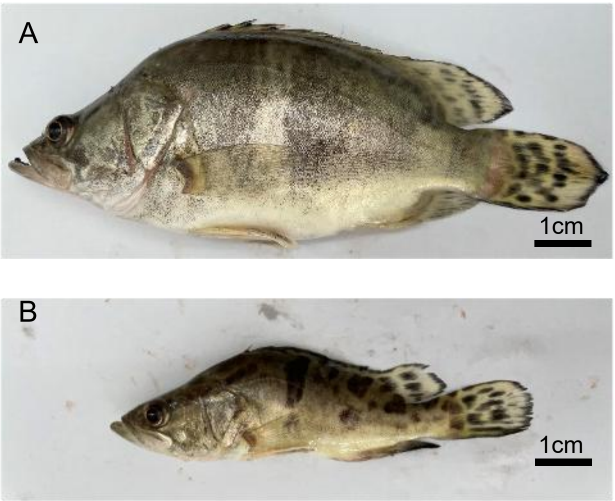

Figure S4. Representative gross appearance of *Siniperca chuatsi* from the same batch following formulated-diet adaptation. (A) An individual with high feed-adaptation performance; (B) an individual with low feed-adaptation performance. Both individuals were selected from the same batch and reared under identical conditions.
